# Multifunctional Melanin Nanoparticles: Synthesis, Characterization, and Magnetic Resonance Imaging-Guided Biomedical Applications

**DOI:** 10.3390/ma19163473

**Published:** 2026-08-17

**Authors:** Khawaja Faheem Shahid, İdil Seçkin, Işıl Tulca Aktürk, Sarra Tarek Hanish, Bugra Ayan, Gizem Kaleli-Can, Mustafa Kemal Ruhi, Engin Baysoy

**Affiliations:** 1 Department of Biomedical Engineering, School of Engineering and Natural Sciences, Bahçeşehir University, İstanbul 34353, Türkiye; idilseckin@outlook.com.tr (İ.S.); engin.baysoy@bau.edu.tr (E.B.); 2 Department of Cardiothoracic Surgery, Stanford University, Stanford, CA 94305, USA; 3 Department of Biomedical Engineering, İzmir Demokrasi University, İzmir 35140, Türkiye; 4 Institute of Biomedical Engineering, Boğaziçi University, İstanbul 34342, Türkiye; 5 Center for Targeted Therapy Technologies (CT3), Boğaziçi University, İstanbul 34342, Türkiye

**Keywords:** melanin nanoparticles, polydopamine nanoparticles, photothermal therapy, photodynamic therapy, drug delivery, magnetic resonance imaging, theranostic applications

## Abstract

Melanin nanoparticles (MNPs) have attracted growing attention in biomedical fields due to their wide bioavailability, high biocompatibility, biodegradability, and multifunctionality. Key features of MNPs are broad-spectrum light absorption, high drug-loading capacity, near-infrared light-triggered drug release and reactive oxygen species generation, efficient hepatobiliary, renal clearance, and superior binding affinity towards metal ions that enhance MNPs’ traceability in magnetic resonance imaging (MRI). Despite challenges such as agglomeration in time, lack of extraction standardization, and a limited number of animal and clinical studies, the strong photothermal conversion and reactive species production abilities of MNPs make them effective photosensitizers and photothermal agents, while their dual capability for molecular binding and metal chelation positions them as promising agents for theranostic applications. This review provides a comprehensive overview of MNPs, covering their structural features, manufacturing methods and associated limitations, and recent advances in their physicochemical characterization. Special focus is given to MRI-guided biomedical applications, including drug delivery, photothermal therapy, and photodynamic therapy, along with critical challenges related to the clinical translation and commercialization of MNP-based platforms. By consolidating recent advances, this review highlights key structure–function relationships that underpin emerging melanin-based nano-systems.

## 1. Introduction

Melanin is an abundant natural biopolymer that is widely distributed across organs and tissues such as. the skin, hair, eyes, mucosal membranes, and brain, along with various living organisms, including fungi, bacteria, plants, and animals [1]. In their natural nanoparticulate form, melanin nanoparticles (MNPs) show diverse physicochemical properties, including broadband ultraviolet–visible absorption, efficient stimuli responsive photothermal conversion and reactive oxygen species generation, intrinsic photoacoustic activity, free radical scavenging, antioxidation capacity, metal ion, and biomolecule chelation [2]. Recently, MNPs and their synthetic analog, polydopamine (PDA), have gained significant attention among researchers due to their intrinsic biocompatibility, biodegradability, chemical versatility, and ease of functionalization [3]. The combination of broad photothermal responsiveness, high affinity towards metal ion chelation (e.g., Fe^2+^, Mn^2+^, Cu^2+^, and Zn^2+^), and efficient loading of biomolecules and drugs renders melanin-based nanoparticles a versatile platform for MRI, therapeutic delivery, and integrated theranostic applications [4,5].

Despite these benefits, it is still challenging to develop exact structure–function connections for melanin-based materials. Due to the participation of radical-mediated polymerization processes and the interaction between covalent crosslinking [6] and noncovalent supramolecular assembly, their chemical architecture and formation mechanisms remain incompletely understood [7]. Therefore, rather than uniform polymers, melanin-based materials are more precisely described as heterogeneous supramolecular ensembles [8], resembling structurally disordered systems such as humic substances and amorphous π-conjugated carbon frameworks [9,10]. Their distinctive broadband optical absorption resulting from disordered π-conjugated systems and amorphous semiconducting behavior, is caused by structural complexity that allows for effective photothermal conversion via non-radiative decay routes [11,12,13,14,15]. Moreover, their surface functioning permits effective loading and regulated release of drugs, while their capacity to chelate paramagnetic metal ions enhances MRI visibility [16,17,18,19].

These distinctive properties make melanin characteristic outcomes put melanin-based systems in a promising position for multimodal and theranostic applications. Melanin nanoparticle-based systems have significant advantages in biomedical imaging, therapeutic interventions and theranostic applications owing to their broad spectral optical absorption, high efficiency of converting near infrared light to thermal energy, intrinsic paramagnetic features and biocompatibility. Their optical and magnetic properties allow them to serve as external contrast agents in photoacoustic imaging while simultaneously providing enhanced T1-weighted magnetic resonance images. Moreover, the generated thermal energy can be used for selective targeting of neoplasms because photothermal treatments are highly target specific.

**Figure 1:**
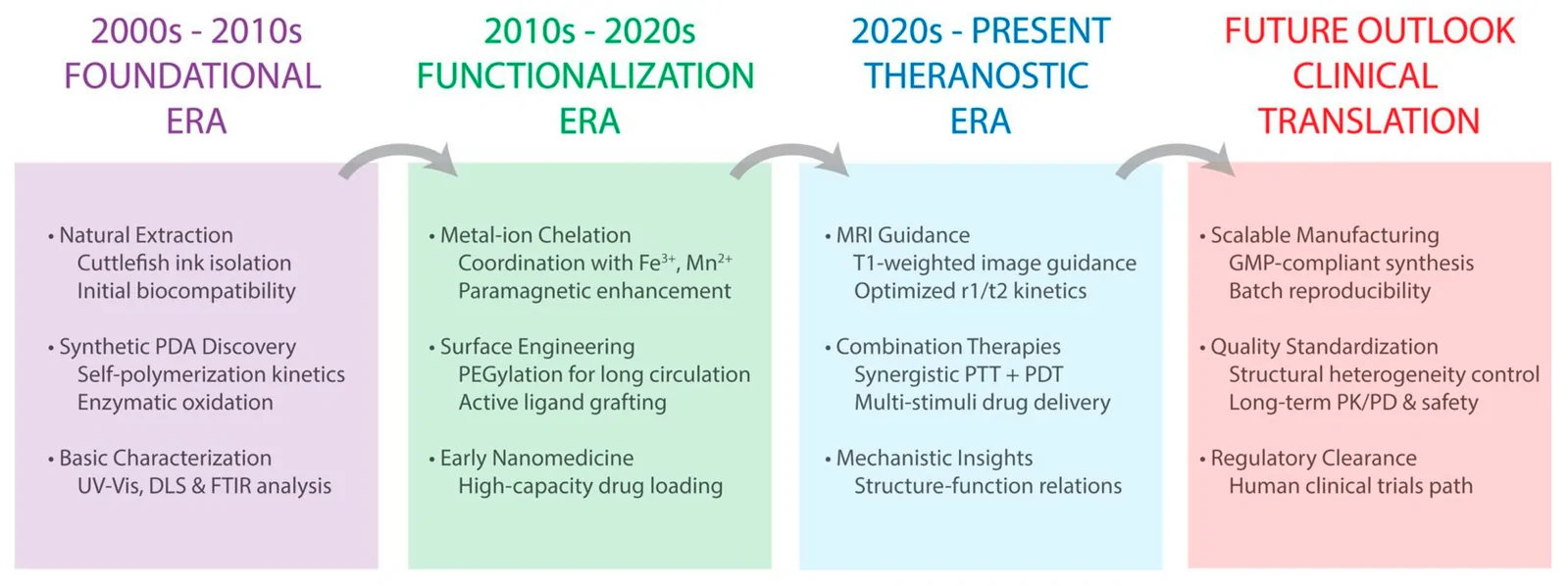
Developmental phases of research related to melanin nanoparticle-based systems for MRI-guided biomedical applications in each decade. The abbreviations used in Figure 1 are MRI, magnetic resonance imaging; PDA, polydopamine; PEG, polyethylene glycol; PTT, photothermal therapy; GMP, good manufacturing practice; and PK/PD, pharmacokinetics/pharmacodynamics.

Zhang et al. (2018) [20] developed PEGylated melanin-conjugated nanoliposomes that directly reach neoplastic tissue to perform combination photothermal therapy guided by photoacoustic and magnetic resonance imaging. The formulation demonstrated consistent photoacoustic signals, strong T1-weighted magnetic resonance contrast, improved photothermal efficiency relative to free melanin, and complete tumor ablation in a mouse model of breast cancer. They concluded that encapsulating melanin in PEGylated liposomes directly enhances the physiological stability and biosafety of the system. Therefore, melanin nanoparticle-based systems can simultaneously serve as diagnostic probes, therapeutic monitoring agents, and platforms for photothermal therapy as shown in Figure 1.

This review provides a comprehensive overview of both MNPs and PDA, with a particular focus on their synthesis, characterization and biomedical applications, including PTT, PDT, and drug delivery with MRI guidance. Advanced techniques are employed for the characterization of MNP structure and composition in terms of particle size, maximum ratio of bonded metal (per MNP or mg of MNPs), stability of metal ion bound, zeta potential (mV), and in vitro and in vivo cytotoxicity. Their clearance pathways in in vivo models are also evaluated. In addition, parameters such as r1 and r2 relaxivity (1/T1 and 1/T2 rate) (mM^−1^ s^−1^) for MRI signal enhancement, photodynamic activity, and drug loading and release behaviors under varying concentrations and light irradiation conditions are assessed. The therapeutic performance of MNPs in various formulations is further evaluated for PTT, PDT, and drug delivery with MRI-based monitoring. This review highlights current challenges and outlines future directions for the clinical translation of melanin nanoparticle-based theranostic platforms with MRI guidance. Unlike previous general reviews on melanin nanoparticle-based nano-systems, this narrative review summarizes the synthesis, structural features, characterization techniques, and biomedical applications (focusing on PTT, PDT, drug delivery, and MRI guidance) of natural melanin nanoparticles (MNPs) and synthetic polydopamine (PDA).

## 2. Methodology/Search Strategy

The present narrative review focuses on theranostic studies in which melanin-based nanoparticles were employed to combine magnetic resonance imaging (MRI) with therapeutic approaches. To identify relevant publications, only peer-reviewed articles published in English were included, whereas conference abstracts and non-peer-reviewed literature were excluded. Study selection was carried out through an initial screening of titles and abstracts, followed by full-text assessment of potentially eligible articles. In addition, the reference lists of the selected publications were examined to identify any further relevant studies. The literature search was conducted using the PubMed, Scopus, and Google Scholar databases without imposing any restrictions on publication date. Search terms included combinations of “natural melanin nanoparticles,” “polydopamine,” “MRI,” “photodynamic therapy (PDT),” “photothermal therapy (PTT),” “drug delivery,” “cancer therapy,” and “theranostics.”

## 3. Synthesis and Isolation of MNPs

Melanin is a high-molecular-weight, irregular, and amorphous biopolymer formed through the oxidative polymerization of indole and phenolic precursors such as L-tyrosine or L-DOPA. It is composed of graphite-like planar units that spontaneously assemble in a hierarchical manner, giving rise to colloidal aggregates that may reach several hundred nanometers in diameter [21,22]. These natural colloidal aggregates can be isolated from a range of natural sources, including cuttlefish ink, black sesame seeds, and animal hair through source-specific multistep extraction and purification protocols [23]. Most of the researchers benefit from cuttlefish (“sepia officinalis”) ink as a conventional extraction method that is based on ultracentrifugation and aqueous washing, without using chemicals. Alternatively, various extraction techniques have been proposed, most commonly alkali-soluble acid precipitation extraction, enzymatic extraction, ultrasonic-based extraction and microwave-based extraction as given in the Table 1 [5]. Of these, alkali-soluble acid precipitation is the most common protocol in the literature due to its operational simplicity, although exposure to strong alkaline and acidic conditions may impair melanin structure and may affect scalability and environmental sustainability adversely [24,25]. Enzymatic extraction has emerged as an alternative method capable of preserving melanosome morphology and yielding more uniform particles through the digestion of proteins tightly associated with melanin, thereby improving dispersion stability and purity [26]. In addition, assisted extraction approaches, such as ultrasonic- and microwave-assisted extraction have been increasingly studied to enhance extraction efficiency, reduce process time, and solvent consumption. Nevertheless, these approaches may also introduce structural alterations, including particle deformation or nonuniform heating effects [27,28]. Even when the same biological source is used, variations in extraction and purification protocols can lead to considerable variability in the physicochemical properties of isolated melanin and, consequently, in the characteristics of the resulting colloidal structures. The amorphous and heterogeneous nature of melanin further complicates controlled extraction and often compromises preservation of its native structural features [2,29,30]. To address these limitations, recent strategies increasingly aim to minimize harsh extraction conditions, preserve functional chemical characteristics, and employ synthetic or biomimetic alternatives such as polydopamine (PDA), which provide improved reproducibility and process control.

PDA has emerged as a well-established synthetic melanin-like material, typically synthesized through the spontaneous oxidative polymerization of dopamine under mildly alkaline conditions in the presence of dissolved oxygen. By mimicking key features of natural melanogenesis, including the generation of indole intermediates such as DHI and DHICA, PDA has been widely used as a synthetic model for investigating melanin-like structure and functions [31]. In addition to covalent oxidation and crosslinking, its formation is also driven by supramolecular assembly under noncovalent interactions, including hydrogen bonding, π−π stacking, and van der Waals interactions [32]. Several methods, such as solution-phase oxidation, enzyme-mediated oxidation, and electro polymerization, can be used to obtain PDA, the details are shown in Table 1 [16]. Because of its ease of use and scalability, solution oxidation in mildly alkaline conditions, where dopamine undergoes spontaneous oxidation and self-polymerization in the presence of dissolved oxygen, is the most popular of these. While electro polymerization allows for the targeted deposition of PDA films on conductive substrates with adjustable thickness, other techniques, like enzymatic oxidation employing tyrosinase or laccase, provide more control over reaction paths [26].

## 4. Structural Organization and Formation Mechanism

Examination of melanin-based system’s structural organization and formation mechanism will enable understanding of their functional behavior. Melanin exists primarily as an irregular, high-molecular-weight, and amorphous biopolymer formed through the oxidative polymerization of phenolic and indole precursors such as L-tyrosine or L-DOPA. Structurally, it is broadly categorized into eumelanin (dark, sulfur-free, indole-based) and pheomelanin (yellow-red, sulfur-containing, benzothiazine-based), alongside specialized biological variants like neuromelanin and pyomelanin. Rather than possessing a rigid, regular covalent polymer backbone, melanin and synthetic polydopamine (PDA) are recognized as heterogeneous supramolecular ensembles resembling structurally disordered systems. Modern biophysical evidence supports a hierarchical noncovalent self-assembly model governed by extended π−π stacking interactions between planar aromatic units (such as 5,6-dihydroxyindole [DHI] and 5,6-dihydroxyindole-2-carboxylic acid [DHICA]) and extensive intermolecular hydrogen-bonding networks. In mixed melanogenesis or neuromelanin architectures, structural organization often conforms to a “casing model,” wherein a reactive pheomelanin core is encapsulated by a photoprotective, electron-scavenging eumelanin surface layer. Furthermore, recent advancements in nanoparticle synthesis reveal that the oxidative self-polymerization of precursors like dopamine does not yield a single uniform product; rather, it follows a dual pathway that simultaneously generates low-density, poorly branched soluble polymers alongside densely crosslinked, spherical nanoparticles driven by supramolecular self-assembly [33]. This flexible, conformationally loose supramolecular architecture provides an exceptionally high surface area and abundant active sites (such as catechols, quinones, and carboxyl groups). These functional groups readily accommodate guest molecules for targeted drug delivery and provide high-capacity chelation sites for paramagnetic transition metal ions (${\mathrm{Mn}}^{2+}$, ${\mathrm{Fe}}^{3+}$, ${\mathrm{Cu}}^{2+}$) without collapsing the underlying framework. Consequently, this unique structural organization directly dictates melanin’s broadband UV-vis semiconductor-like absorption, non-radiative thermal dissipation for photothermal therapy (PTT), and high $r_{1}$-relaxivity performance in magnetic resonance imaging (MRI) guidance [34].

## 5. Characterization Techniques

Revealing the structural features of melanin nanoparticles is essential to improve their functional performance in biomedical application. A wide array of analytical techniques has been employed to characterize MNPs [11]. Transmission electron microscopy (TEM), scanning electron microscopy (SEM), scanning transmission electron microscopy (STEM), confocal laser scanning microscopy (CLSM), X-ray diffraction (XRD), energy-dispersive X-ray spectroscopy (EDS), X-ray photoelectron spectroscopy (XPS), inductively coupled plasma optical emission spectroscopy (ICP-OES), inductively coupled plasma mass spectrometry (ICP-MS), inductively coupled plasma atomic emission spectroscopy (ICP-AES), Fourier transform infrared spectroscopy (FTIR), dynamic light scattering (DLS), liquid chromatography (LC), infrared thermal imaging, and flow cytometry are the commonly used methods for the characterization of MNPs [11].

XRD distinguishes crystalline structures by analyzing X-ray diffraction patterns, revealing phase composition changes in MNPs [11]. EDS confirms elemental composition and distribution, which is critical for understanding metal ion coordination and nanoparticle stability [35]. CLSM provides three-dimensional imaging to evaluate cellular uptake and endosomal escape, key for understanding drug delivery mechanisms, by using a focused laser beam to illuminate samples [36].

TEM and SEM deliver high-resolution images that reveal particle morphology and size distribution, confirming that MNPs generally exhibit uniform spherical shapes post-extraction [37,38]. DLS measures particle size distribution and zeta potential through analysis of scattered light intensity fluctuations, providing insight into colloidal stability and biological interactions [39]. For example, the NanoZetasizer system has been used to analyze the hydrodynamic diameter and zeta potential of PEGylated MNPs, monitoring physiological stability by tracking size changes in serum and PBS [40].

MNPs have an inherent size depending on the source. Coating with PEG (polyethylene glycol) or subsequent binding with metal ions increases the hydrodynamic size and overall size slightly [41]. In contrast, PDA can be engineered to a wider range of sizes, from small (~10 nm) to larger sizes of around 100–150 nm or even over 300 nm when loaded with drugs [42,43,44].

Recent studies have shown that PEGylated MNPs typically exhibit zeta potential values ranging from –6 mV to –30 mV, whereas bare MNPs may display more negative values, often below –40 mV [34,42,43,44,45,46,47,48]. LC, a separation technique for identifying and quantifying components, has been applied to quantify concentrations of drugs, such as Camptothecin (CPT) within formulation [49].

The use of ICP-OES/ICP-MS/ICP-AES quantifies the metal ion content of MNPs. The combinational use of ICP-OES/ICP-MS/ICP-AES and ultracentrifugation determine the stability of metal ion chelation with MNPs in terms of binding and release performance. Studies revealed that MNPs exhibit strong chelation stability with Mn^2+^ ions, achieving binding efficiencies up to 98% [34,43,44]. Similarly, other metal ions, such as Cu^2+^ Fe^3+^, and Gd^3+^, also demonstrate high binding stability with MNPs, typically exceeding 90% [33,40,41]. FTIR spectroscopy plays a key role in characterizing surface modifications of MNPs. For example, PEGylation was confirmed using an iS50 FTIR spectrometer by detecting amine-specific signals through fluorescein labeling, with ethylamine employed as a reference standard [50].

## 6. Biomedical Applications with Melanin Nanoparticle-Based Systems

Melanin nanoparticles have gained significant importance due to their role in advancing both diagnostic and therapeutic strategies, particularly in the field of oncology and interventional radiology, owing to their intrinsic ability to chelate metal ions and biomolecules. The functional advantages of MNPs benefit not only therapeutic applications, such as drug delivery, PTT, and PDT, but also medical imaging modalities like MRI.

### 6.1. MRI-Guided PTT/PDT and Drug Delivery with Melanin Nanoparticle-Based Systems

MRI is a favorable imaging platform due to its X-ray free nature, superior soft tissue contrast, and collection of physiological data like temperature, pressure, diffusion, and perfusion, together with anatomical information. MNPs chelated with paramagnetic metal ions, such as Cu^2+^, Fe^3+^, and Mn^2+^, significantly improve the visibility of MNPs in spin-lattice T1-weighted MR imaging without adversely affecting their therapeutic capabilities, as shown in Table 2.

Similarly, Thirumurugan et al. developed metal–organic framework-derived copper benzene-1,3,5-tricarboxylate conjugated PDA-based (Cu-BTC@PDA) nanowires for the localization and diagnosis of tumors via MRI, offering high photothermal conversion efficiency alongside robust drug-loading and controlled-release properties. The increase in brightness in T1-weighted MR images was correlated with the amount of Cu content, which varied between 62.5 and 1000 µg/mL [51].

Xu et al. further optimized Mn^2+^-chelated PEGylated MNPs (MNP-PEG-Mn^2+^), enhancing biocompatibility and colloidal stability for imaging and therapeutic use [47]. In vitro MRI using a 3.0 T clinical scanner demonstrated (Siemens, Erlangen, Germany) that MNP-PEG-Mn structures provided strong T1-weighted contrast enhancement, indicating superior performance compared to conventional gadolinium-based agents [47]. Furthermore, in vivo MR imaging in tumor-bearing mice showed effective MNP accumulation in the tumor region following intravenous injection, with MRI signal intensity peaking at 3 h post-injection and gradually diminishing over 5 days, consistent with nanoparticle clearance kinetics [47]. The integration of advanced synthetic strategies, surface modification, and multimodal imaging facilitates precise tumor targeting and monitoring, advancing MNPs and PDA toward clinical translation for cancer therapy and diagnosis.

The data given in Table 2 indicates that both MNPs and melanin-mimicking polydopamine nanoplatforms are promising for theranostic applications guided with MRI. Even though PEGylation and metal ion chelation does not increase the overall size of MNPs significantly, drug-loaded and polymer-coated MNPs and polydopamine-based systems tend to be larger. This outcome is critical because smaller nanoparticles are more appropriate for tissue penetration and renal clearance, while larger particles may bind with therapeutic agents and may enable tumor accumulation through retention effect. Findings related to r1 relaxivity values underpin the requirement of metal ion chelation for satisfying the MRI trackability of MNPs and polydopamine.

T1-weighted spin echo or gradient echo are the most common MR scanning sequences for adequate CNR. Since MR images are acquired using different magnetic field strengths (0.5 to 11 T) and varied metal loading, direct comparison among the melanin-containing formulations is not possible in terms of CNR and relaxivity rates. Most melanin-based systems have high viability at concentrations up to 800 μg/mL, while PEGylation and silica coating reduce toxicity and improve colloidal stability. In vivo results of the studies have confirmed the contrast enhancement properties of metal ion-chelated melanin platforms, with effects that appear between 0.5 and 24 h after administration, supporting the clinical potential of melanin-based platforms as tumor-imaging agents.

Renal and hepatobiliary pathway clearance occurred through the liver, kidneys, and spleen within five days. Although no substantial tissue damage, inflammation, or behavioral changes were reported after histological examination, these results should be interpreted cautiously, and more detailed long-term pharmacokinetic and toxicity assessments are required. Rather than serving solely as diagnostic agents, drug-loaded melanin-based platforms combine chemotherapeutic effects with MR monitorization. In general, melanin-based formulations have exhibited concentration and particle size-dependent thermal responses, making them suitable for PTT, PDT and stimulus-responsive drug delivery applications.

Most nanoplatforms were activated using 808 nm near-infrared irradiation, consistent with the optical characteristics of the melanin nanoparticles in the literature. Some studies have shown improved therapeutic efficacy by combining photothermal treatment with drug delivery, which may minimize the required drug dose. However, all nanoplatforms should be optimized considering tumor accumulation, irradiation conditions, and drug loading/release performance.

### 6.2. Melanin Nanoparticle-Based Applications with Photodynamic and Photothermal Therapies

PDT and PTT have emerged as powerful minimally invasive modalities for cancer treatment. PDT operates by generating ROS, particularly singlet oxygen, via the excitation of photosensitizers in the presence of molecular oxygen upon light exposure, selectively inducing cytotoxicity in tumor cells while minimizing harm to normal tissue [48,51,52,53]. PTT, on the other hand, employs photo-absorbing materials such as gold or carbon nanoparticles to convert absorbed NIR light into localized hyperthermia, resulting in effective ablation of cancer cells [48]. The efficacy of both therapies strongly depends on the optical properties of the agents or nanomaterials used. MNPs, owing to their broad absorption spectrum and intrinsic PDT/PTT activity, have demonstrated promise as dual-functional therapeutic agents. Although MNPs exhibit maximum absorbance in the region between 200 and 400 nm, their photothermal performance remains robust even at longer wavelengths such as 808 nm, used in clinical settings [21,54]. For example, MNP dispersions at 200 µg/mL have been reported to achieve photothermal conversion efficiencies as high as 87.65% under 808 nm NIR laser irradiation [48]. Furthermore, studies have shown that MNP-containing formulations can effectively induce ROS generation under UV or NIR irradiation. Specifically, singlet oxygen production significantly increased upon irradiation with 400 nm and 670 nm in MNP-based systems [48,55].

Chen et al. systematically investigated the r1 relaxivity values of MNP-PEG chelates with Gd^3+^, Mn^2+^, Fe^3+^ and Cu^2+^ using 1.5 T (in vitro) and 3 T (in vivo) clinical MR scanners [33]. R1 relaxivity (1/T1 rate) values were measured as MNP-PEG-Fe: 11.1 mM^−1^ s−1, MNP-PEG-Gd: 61.9 mM^−1^ s^−1^, MNP-PEG-Cu: 9.7 mM^−1^ s^−1^, MNP-PEG-Mn: 48.7 mM^−1^ s^−1^, respectively. All the solutions presented better r1 relaxivity compared to commercial Gd-DTPA (5.978 mM^−1^ s^−1^), while MNP-PEG-Gd provided 10 times higher value (61.853 mM^−1^ s^−1^) when the loading mass ratio was Gd3+: MNP = 1:1 [34]. The same group engineered polymer-encapsulated PDA nanocomplexes (PDAs@CPx), demonstrating both a strong T1/T2 dual-mode MRI contrast agent with high longitudinal and transverse relaxivity and robust PTT/PDT functionality [56].

The duration of irradiation plays a critical role in PTT efficacy. For instance, a 6 min exposure at a power density of 1.31 W/cm^2^ has been shown to induce significant photothermal effects in HeLa cells [56]. Additionally, the type of cancer cell line used in PTT/PDT studies can influence treatment outcomes. The performance of MNP-based PTT/PDT approaches have been evaluated in various cell lines, including human larynx epidermal carcinoma (Hep-2), human colorectal carcinoma (HCT 116), mouse embryonic fibroblast (NIH 3T3), cervical cancer (HeLa), breast cancer (4T1), lung carcinoma (A549) and human liver cancer (HepG2), as summarized in Table 2.

Lui et al. investigated the photothermal performance of dopamine–melanin colloidal nanospheres (Dpa-melanin CNSs) in 4T1 and HeLa cells. Upon 808 nm laser irradiation at 2 W/cm^2^ for 500 s, aqueous CNS dispersions at 200 μg/mL exhibited a temperature increase of 33.6 °C, sufficient to raise tumor temperatures above 50 °C, enabling effective decreases in tumor volume in 4T1 tumor-bearing mice [44]. These CNSs demonstrated biodegradability in the presence of endogenous hydrogen peroxide, indicating potential for in vivo clearance [44].

### 6.3. Melanin Nanoparticle-Based Systems in Drug Delivery Applications

MNPs have emerged as a versatile platform for drug delivery applications due to their multifunctional surface chemistry. The abundance of catechol, amine, quinone, and imine groups on their surface enables drug loading through π−π stacking, hydrogen bonding, covalent conjugation, or physical encapsulation [4]. Photosensitizers like indocyanine green, antibiotics, and anti-inflammatory compounds [17]. Moreover, their ability to chelate paramagnetic metal ions such as Gd^3+^, Mn^2+^, Fe^3+^, and Cu^2+^ allows MNPs to serve as contrast agents, particularly for T1-weighted MRI, where they provide a positive contrast [34]. Co-functionalization with metal ions and therapeutic or targeting molecules further enhances their diagnostic and therapeutic versatility, establishing MNPs as strong candidates for theranostic applications [34]. Due to their favorable biocompatibility and biodegradability, MNPs can undergo controlled degradation in response to ROS, such as hydrogen peroxide (H_2_O_2_) and singlet oxygen, enabling environmentally triggered release mechanisms [4,55]. Their colloidal stability can be further optimized via surface modification, promoting dispersion in physiological media. However, to support clinical translation, systematic evaluation of their long-term toxicity, pharmacokinetics, and the standardization of synthesis and applications protocols in clinics remain essential [4].

For example, Gd-Mel@SiO_2_ nanoparticles have demonstrated high drug-loading efficiency, approximately 70%, enabling a substantial therapeutic payload and controlled release profile [46]. Such prolonged release helps maintain therapeutic drug concentrations at the tumor site over extended durations, while reducing systemic toxicity. Moreover, the nanoscale dimensions and favorable surface properties of MNPs result in efficient cellular uptake, a key determinant of successful drug delivery. Typically exhibiting a negative zeta potential, MNPs minimize opsonization and protein adsorption during circulation, thereby extending systemic half-life and facilitating tumor accumulation via the enhanced permeability and retention effect [34,42,43].

### 6.4. Combination of PTT/PDT and Drug Delivery with Melanin Nanoparticle-Based Systems

The integration of PTT and PDT with MNP-based drug delivery systems to offer a synergistic strategy for cancer treatment is an active area of research since the generation of localized heat or ROS through light exposure can enhance drug release kinetics, facilitate deeper tissue penetration, and promote tumor-specific accumulation. For example, the PDAs@CP3-DOX nanocomplex exhibits NIR-responsive drug release, where heat generated by PTT triggers doxorubicin (DOX) release, thereby improving cytotoxic effects against cancer cells [56].

In vitro HeLa cells confirmed enhanced apoptosis following combined treatment, as evidenced by flow cytometry and live/dead staining analyses. Similarly, PheoA-functionalized MNPs showed increased DOX release (~23%) after 30 min of NIR irradiation, compared to ~5% without irradiation, highlighting the role of photothermal effects in modulating drug–nanoparticle interactions [57]. Therefore, MNPs have been successfully employed to deliver a broad range of therapeutic agents, including chemotherapeutics such as doxorubicin [58], paclitaxel, cisplatin [59]. 

Copper-based PDA nanocomposites (CuPDA NPs) also exhibit favorable pharmacokinetics, including prolonged circulation and improved excretion profiles, which are critical for clinical translation [41]. In vivo studies with CPT-loaded PDA nanoparticles demonstrated high photothermal stability and the ability to induce ferroptosis in lung cancer models [48]. In BALB/c nude mice, NIR exposure of tumors led to significant local temperature increases, confirming the efficacy of combined chemo-PTT strategies [46].

Kang et al. developed a multifunctional nano-system by integrating Mn^2+^-chelated melanin-like poly(L-DOPA) nanoparticles with the photosensitizer PheoA, chemotherapeutic agents (doxorubicin or SN38), and the targeting ligand folic acid, aiming for a synergistic platform combining image-guided photothermal, photodynamic, chemotherapeutic, and targeted cancer therapy [48]. Upon 808 nm NIR laser irradiation (1.2 W/cm^2^, 20 min), the system reached a local temperature increase of 52 °C and exhibited a photothermal conversion efficiency of 87.65% at a concentration of 200 µg/mL in HCT116 cells [48]. In parallel, the system demonstrated potent PDT activity, under 670 nm laser irradiation (150 mW/cm^2^ for 10 min), significant singlet oxygen generation was observed, resulting in effective cellular apoptosis [48]. This system also retained high drug-loading capacity, with a maximum DOX payload of 35% [59,60,61,62]. Triggered drug release studies showed that DOX release reached ~23% after 30 min of NIR irradiation, depending on exposure time and power density (0.5–2.0 W/cm^2^). As summarized in Table 3, such multifunctional MNP- or PDA-based nano-systems hold great promise for enhancing therapeutic efficacy by integrating PTT, PDT, chemotherapeutic, and targeting functionalities into single-agent modality treatments.

Recent advances in biomedical research also benefit multimodal imaging-guided therapy to improve precision in diagnosis and targeted treatment. Cho et al. presented silica-coated Gd^3+^ chelating-melanin nanoparticles (Gd-Mel@SiO_2_ NPs) for image-guided prostate cancer PTT. Gd-Mel@SiO_2_ NPs were not only used for acquiring MR images with high spatial resolution but also employed for fluorescent imaging to enhance real-time tracing and delivery of photothermal ablation on the tumor surface [46]. Another study conducted by Ju et al. benefits from synergistic imaging of MRI and Raman imaging for breast carcinoma cells using hollow gold nanoparticles (h-Au) encapsulated and complexed with PEG and Fe^3+^ ion MNPs [63,64]. Together with the T1-weighted MRI contrast-enhancing capability of MNPs, Raman imaging provided visualization of the cellular-based spectroscopic activities of MNPs [65].

In recent years, Baysoy et al. have focused on developing a novel method that employs MNPs to improve the visibility of MRI-compatible interventional instruments in MR images. Initial study results of Baysoy et al. have shown that MNP-filled catheters and MNP-coated surgical guidewires using dip coating and electrospinning have great potential for fabrication of MRI-safe and trackable invasive medical devices that can be used for interventional MRI procedures, especially for vascular disease treatment [66,67,68,69].

## 7. Cytotoxicity of Melanin Nanoparticles

Assessing the cytotoxicity of MNPs is crucial for ensuring their safe use in the human body. MNPs are often functionalized with metal ions such as Fe^3+^, Gd^3+^, Cu^2+^, and Mn^2+^ to enhance their diagnostic capabilities under MRI, while PEGylation improves their stability and circulation time in vivo. Particularly, both natural and modified MNPs have demonstrated low toxicity and good biocompatibility in various in vitro and in vivo studies, as shown in Table 3.

For example, PEGylated MNP-metal ion complexes (MNP-PEG-Fe/Gd/Cu/Mn) were evaluated on H9c2 rat cardio myoblast cells at concentrations up to 800 µg/mL under standard culture conditions (5% CO_2_, 37 °C) for 24 h. Corresponding in vivo studies involved intravenous administration of 1 mg MNPs to BALB/c mice, with examination of heart, liver, and spleen tissues daily for up to 30 days post-injection. Both acquired MR images and ICP-MS results of metal ion content after sacrifice of the mice revealed that MNPs were mainly cleared through the kidney and liver, and no negative change was observed in terms of biosafety [34].

In another study, PEGylated Fe^3+^-chelated melanin-like nanoparticles (MelNPs) did not indicate acute cytotoxicity or morphological changes when they were incubated with HeLa cells at concentrations up to 200 µg/mL and assessed using WST-1 assays for 24 h [40]. Accordingly, after administration of PEGylated Fe^3+^-chelated MelNPs to a mouse, a positive signal enhancement in the spleen and the liver were observed within 1.5 h in T1-weighted MRI images. Enhanced contrast following the injection disappeared after 24 h, proving clearance from all organs.

Fan et al. showed that PEGylated Fe^3+^-chelated MelNPs demonstrated negligible cytotoxicity up to 3125 µg/mL in NIH-3T3 and U87MG cells measured via MTT assays [43]. Manganese-chelated MNPs (MNP-Mn^2+^) have also been studied for MRI applications. In vitro incubation of Hep-2 and NIH-3T3 cells with MNP-Mn^2+^ at concentrations up to 1600 µg/mL resulted in negligible cytotoxicity [43,44,45,46,47].

PDA-based nanostructures have also been widely explored due to their inherent biocompatibility and metal-chelating capacity. Rui et al. investigated copper-loaded PDA (CuPDA NPs) on 293, KB, and HeLa cell lines, reporting >70% cell viability even at concentrations up to 400 µg/mL after 24 h [41]. Biodistribution studies indicated accumulation in the liver, spleen, and kidneys, yet no signs of hepatic or renal dysfunction were observed within 24 h, and histological evaluations showed no tissue damage or inflammation over a 30-day period post-administration [41]. Chen et al. synthesized iron-chelated PDA nanoparticles -(PDAs@CP3) and assessed cytotoxicity in HeLa cells via 24-h MTT assays, confirming low toxicity [56]. Complementary in vivo studies in BALB/c mice revealed no pathological changes in major organs up to 30 days post-injection, supporting good systemic biosafety [56]. Similarly, dopamine–melanin colloidal nanospheres (Dpa-melanin CNSs) demonstrated favorable in vitro biocompatibility, maintaining ~90% cell viability in 4T1 breast cancer cells at concentrations up to 1.2 mg/mL [44].

In another study, pheophorbide a (PheoA)-decorated poly(L-DOPA) nanoparticles were tested on HCT-116, and HEY-T30 cells using WST assays and exhibited no significant toxicity up to 100 µg/mL [48]. Corresponding in vivo analyses showed preserved tissue morphology and absence of tumor necrosis [48]. CPT-loaded and manganese dioxide (MnO_2_)-coated PDA systems were evaluated in A549 and LLC lung cancer cell lines using MTT assays, with blank PDA NPs demonstrating >75% viability at 20 µg/mL. In vivo imaging confirmed drug retention up to 48 h, and metabolic clearance via the liver and kidneys occurred without observable toxicity [45]. Moreover, PDA-functionalized metal–organic framework nanowires (Cu-BTC@PDA) retained high biocompatibility in HepG-2 liver cancer cells across a concentration up to 100 µg/mL over 24 h, further demonstrating the biosafety of PDA-integrated nano-systems [52].

As highlighted in Table 2, a broad spectrum of in vitro studies has demonstrated the negligible cytotoxicity of both metal-chelated MNPs and PDA structures across various cell lines, including H9c2, HeLa, NIH 3T3, U87MG, HCT116, 4T1, A549 and HepG2. These nanoplatforms have maintained high cell viability at concentrations of up to milligrams per milliliter, even following prolonged exposure. Moreover, diverse formulations such as PEGylated MNP–metal ion complexes, MNP-Mn^2+^ systems, CuPDA nanoparticles, and Fe^+3^-chelated PDA demonstrated in vivo compatibility, with no signs of acute or chronic toxicity during observation periods of up to 30 days. These results were supported by behavioral monitoring, weight tracking, and histological examinations, which consistently reported the absence of inflammation, organ damage, or pathological changes [40].

Studies have indicated that various melanin nanoparticle-based formations like PEGylated, metal ion-complexed, and drug-loaded systems exhibit low acute cytotoxicity and enable high cell viability after 24 h of exposure [41]. In general, cytotoxicity performance of melanin nanoparticle-based systems depends on nanoparticle size, composition, coating, metal ion chelation rate, drug loading, concentration, and cell type [42]. Due to the lack of in vivo studies, major behavioral, biochemical, or histopathological abnormalities during short-term observation are missing in the reports. Therefore, the current results underpin short-term biocompatibility of melanin nanoparticle-based platforms rather than their standardized long-term biosafety [43,44,45,46].

## 8. Challenges in Clinical Translation and Commercialization

Owing to MNPs’ and PDA’s unique physicochemical and biological properties, including broadband optical absorption, photothermal conversion capability, radical scavenging activity, metal ion chelation, and intrinsic biocompatibility, these nanomaterials have been accepted as promising candidates for a wide range of biomedical applications [19]. However, despite extensive preclinical progress of melanin-based materials in the literature, several critical challenges continue to limit their clinical translation and commercialization. While melanin itself and melanin-related biological pathways have reached clinical relevance, melanin-based nanoplatforms remain largely confined to preclinical investigation [70,71]. The formation of well-defined chemical structures and reproducible material properties remains challenging because melanin is produced through complex polymerization pathways and hierarchical supramolecular assembly processes. As a result, achieving consistent material performance across different studies is not straightforward, and this structural complexity also complicates regulatory evaluation. Another major challenge is the lack of standardized and scalable manufacturing protocols. Variations in extraction methods, precursor selection, reaction conditions, and post-synthesis processing can lead to substantial batch-to-batch differences in particle size, surface chemistry, metal-binding capacity, and drug-loading performance. Such variability makes it difficult to directly compare results across studies and remains a significant obstacle to quality control, large-scale manufacturing, and future Good Manufacturing Practice (GMP)-compliant production. Establishing standardized synthesis, characterization, and quality-control workflows will therefore be essential for improving reproducibility and facilitating the clinical translation of melanin-based nanoplatforms [26].

Despite increasing patent activity and strong preclinical evidence, a significant gap remains between research and clinical implementation [72,73]. No melanin-based nanoparticle system has advanced to clinical trials yet, and most research has only conducted small experiments using in vitro and early in vivo models [74]. Such a gap underlines the necessity of more thorough preclinical validation and study designs that are applicable to current clinical practice.

## 9. Discussion

The integration of melanin nanoparticles with metal ions and drug delivery systems, whether alone or in combination with PTT/PDT, presents a comprehensive and adaptable strategy for cancer therapy [44,45,46,48,51,62]. Parameters such as drug loading, stimuli-responsive drug release kinetics, photothermally enhanced cellular uptake mechanisms, excretion pathways, irradiation wavelength and duration, and the choice of target cell type all collectively determine therapeutic outcomes and safety profiles.

Agglomeration is another important determinant of the multifunctional performance of organic nanoparticles. It can affect their effective hydrodynamic size, morphology, and interactions with biological components. These changes can also influence their optical properties, which are relevant to the functionality of organic nanoparticles [75,76]. For example, Zhang et al. investigated aggregation-caused quenching in fluorescent organic materials and developed AIEgen-containing nanoparticles to maintain fluorescence in the aggregated state [77]. This study demonstrates the relationship between aggregation behavior and the optical properties of organic nanomaterials. In a different context, Morales-Bonilla et al. showed that asymmetric agglomeration of liposome-encapsulated nanodiamonds in blood plasma influenced their photothermal response and the release of encapsulated cargo [78]. Together, these studies illustrate how changes in the aggregation or agglomeration state can affect the optical and functional properties of organic nanomaterials.

The type of chelated metal ion, and the binding ratio of metal ion at MNPs, as well as the concentration of whole MNPs solution are the key parameters to induce higher r1 relaxivity values at MRI. Although increasing the loading mass ratio of metal ions at MNPs enables higher r1 relaxivity values, the relaxivity value reaches a maximum level at a specific level of bonded metal ions [33]. In contrast, signal enhancement effects of the samples containing MNPs are negligible in MRI [33,74]. Therefore, chelation with metal ions is essential for MNPs to serve as an MRI contrast agent in T1-weighted images.

Conjugating with PEG or a drug agent does not cause significant changes in the contrast of metal ion-chelated MNPs under MR imaging [33,39,42]. This property paves the way for visualizing MNP-based nano-systems using MRI without adversely affecting targeted drug delivery applications. Although some research groups have studied T2 maps and measured r2 relaxation times in different concentrations, contrast improvement is not significant in T2-weighted images as much in T1-weighted images in MRI [40,46,56,64].

Accordingly, future research may focus on hybrid systems, like metal ion-chelated and polymer-coated or metal–organic framework-integrated melanin nanoparticles, to enhance drug-loading capacity and photoconversion efficiency while maintaining biodegradability and traceability under MRI. Despite these promising tremendous features, clinical translation faces three major challenges: (1) standardizing synthesis protocols to minimize batch-to-batch variability in metal content (for example, mass ratio of Fe^3+^ or Cu^2+^ in sepia-derived MNPs) and monomer or drug composition (DHI/DHICA/PEG/DOXO ratios); (2) employing advanced characterization techniques such as solid-state NMR and cryo-EM to better understand hierarchical assembly at the nanoscale (10^−9^ to 10^−7^ m) and its correlation with therapeutic performance; (3) conducting more extensive in vivo evaluation in terms of their long-term biodistribution and immunological compatibility remains essential for safe clinical implementation. By uniquely bridging diagnostic and therapeutic modalities, MNPs or PDA-based nano-systems represent a transformative platform for precision medicine.

Despite the remarkable progress made in this field, several important challenges still need to be addressed. One of the most significant is the inherent structural complexity of melanin and melanin-like nanoparticles. Unlike many synthetic nanomaterials with well-defined molecular structures, these materials are formed through complex oxidative polymerization and hierarchical self-assembly processes, resulting in chemically heterogeneous structures. As a result, nanoparticles prepared using seemingly similar synthesis conditions may still differ in their molecular composition, oxidation state, surface chemistry, and metal-binding properties. Such variability makes it difficult to compare findings across studies, limits reproducibility, and poses important challenges for quality control and large-scale manufacturing.

## 10. Conclusions

Melanin nanoparticles and their synthetic analogs, especially polydopamine, represent a promising and versatile platform for advanced biomedical and theranostic applications. These nanoparticles exhibit a unique set of intrinsic properties, such as high biocompatibility, biodegradability, strong broadband light absorption, and a significant chelation affinity for metal ions and drugs. The development of functionalized MNPs has positioned them as multifunctional agents that can simultaneously perform three essential roles for PTT/PDT and drug delivery. Their high biocompatibility, degradability and binding capacity to metal ions present a potential MRI contrast agent especially for T1-weighted MR images that can be used for localization, real-time tracking, and therapeutic monitoring. The intrinsic photothermal and photodynamic properties of MNPs facilitate synergistic cancer treatment when used alongside chemotherapeutic agents, typically employing NIR-generated heat to initiate controlled drug release. Current research indicates strong efficacy, low in vitro toxicity, and favorable in vivo clearance kinetics; however, the clinical translation of MNP-based nano-systems depends on addressing critical challenges. The standardization of synthesis, comprehensive structural characterization through advanced techniques, and thorough long-term in vivo toxicity and efficacy studies are essential subsequent steps to guarantee safety and consistency across batches for clinical application. By addressing these challenges, melanin-based nano-systems are positioned to advance beyond the laboratory, providing a significant and transformative resource for achieving precision in cancer diagnosis and therapy. Nevertheless, realizing this potential will require substantial advances in structural characterization, standardized manufacturing, long-term safety assessment, and clinically relevant validation studies before melanin-based nanoplatforms can become viable therapeutic products.

## Figures and Tables

**Table 1:** Overview of extraction and synthesis methods for natural and synthetic melanin nanoparticles.

Method of Synthesis/Preparation	Nanoparticle Properties	Representative Applications	Advantages	Limitations	Refs.
Aqueous washing and centrifugation	Preserves naturally occurring colloidal/melanosomal structures; particle size and morphology remain source-dependent	PTT, PAI, MRI after metal chelation, drug delivery	Simple; minimal chemical modification, good preservation of native properties	Limited purification when used alone; residual low-molecular-weight impurities; source-dependent heterogeneity	[18,22]
Alkali-soluble acid precipitation	Produces isolated natural melanin that can subsequently form colloidal particles; native supramolecular organization may be altered	Drug delivery, imaging, PTT, antioxidant applications	Simple; inexpensive; broadly applicable to different biological sources	Harsh pH conditions may modify native structure and functional groups; chemical waste; limited reproducibility and scalability	[23,24]
Enzymatic extraction	Better preservation of melanosome morphology and associated structural features; potentially improved purity and dispersion	Biomedical platforms requiring preserved native melanin features	Mild conditions; selective removal of associated proteins, reduced structural damage	Enzyme cost; longer/more complex processing; source-specific optimization; scale-up challenges	[24,25]
Ultrasound-assisted extraction	Enhanced dispersion and mass transfer; may reduce aggregation and facilitate formation of water-dispersible melanin	Antioxidant systems, nanocarriers and subsequent biomedical formulations	Short processing time; enhanced extraction efficiency, reduced solvent use	Excessive sonication may alter particle morphology and supramolecular organization	[26,27]
Microwave-assisted extraction	Rapid matrix disruption and melanin release through volumetric heating	Preparation of melanin for subsequent nanoformulation	Rapid; reduced solvent consumption, potentially high extraction yield	Non-uniform heating and structural alterations; equipment and scale-up requirements	[5]
Solution-phase oxidative polymerization of dopamine	Typically spherical PDA nanoparticles; size and surface properties can be tuned through pH, precursor concentration and reaction conditions	Drug delivery, PTT/PDT, PAI, MRI after metal chelation, theranostics	Simple; reproducible, scalable; high control over particle size and surface chemistry	Material properties strongly depend on synthesis conditions; PDA does not completely reproduce natural melanin structure; polymerization mechanism remains incompletely resolved	[9,16,19,30]
Enzyme-mediated oxidation/electropolymerization	Enzymatic oxidation provides melanin-like structures under mild conditions; electropolymerization generates controlled surface-confined PDA layers	Biomimetic nanomaterials; biosensors and conductive biomedical interfaces	Mild/biomimetic enzymatic route; localized and controllable electrodeposition	Enzyme stability/cost and processing complexity; electropolymerization mainly restricted to conductive substrates rather than free nanoparticles	[16,19,25]

**Table 2:** Characterization of melanin nanoparticle-based systems in terms of MRI visualization and cytotoxicity.

Natural or Melanin-Like (Polydopamine) Nanoparticles	Particle Size (Before and After PEG/Metal Ion/Tumor Targeting Biomarker Binding)	Used MRI or EPR	Maximum MRI Signal Enhancement, r1 Relaxivity (1/T1 Rate) (mM^−1^ s^−1^)	In Vitro Biocompatibility Test/Result	In Vivo MRI and Cytotoxicity Evaluation	Ref.
	MNP/MNP-Metal ion/MNP-PEG/MNP-Drug (nm)					
Natural Melanin Nanoparticles (MNP-PEG-Metal Ion)	MNP-PEG-Fe^3+^: 5.8 MNP-PEG-Gd^3+^: 6.9 MNP-PEG-Cu^2+^: 3.7 MNP-PEG-Mn^2+^: 3.4	In vitro: 1.5 T clinical MRI in vivo: 3 T clinical MRI	MNP-PEG-Fe: 11.1 mM^−1^ s^−1^ MNP-PEG-Gd: 61.9 mM^−1^ s^−1^ MNP-PEG-Cu: 9.7 mM^−1^ s^−1^ MNP-PEG-Mn: 48.7 mM^−1^ s^−1^ (at the loading mass ratios of Gd^3+^:MNP = 1:1, Mn^2+^:MNP = 0.5:1, Fe^3+^:MNP = 0.1:1, and Cu^2+^:MNP = 0.1:1)	Cytotoxicity (in vitro): MNP-PEG-metal ions (0–800 μg/mL) showed no acute cytotoxicity or morphological changes in H9c2 cells after 24 h (CCK-8 assay), indicating promising biocompatibility.	Peak liver/kidney signal at 0.5–2 h with full clearance by 24 h (renal/hepatobiliary); zero hepatic/renal toxicity at 1 mg/mouse after 1/7 days, proving high biocompatibility for MRI.	[34]
Natural Melanin Nanoparticles (Fe^3+^-Sepia Melanin) Melanin-like Nanoparticles (Fe^3+^-MNPs)	MNP-Fe: varied in 98, 318, 570.	In vitro: 3T clinical MRI In vivo: 7T/20 micro-MRI	MNP-Fe^3+^: 17 mM^−1^ s^−1^ (for 98 nm MNP)	Test: HeLa cells (human cervical carcinoma cells) were incubated with MNP-PEG-Fe at levels up to 200 μg/mL and measured using the WST-1 assay for 24 h. Result: No apparent acute cytotoxicity or morphological changes.	T1 signal enhanced in spleen (0.5 h) and liver (1.5 h); liver returned to baseline at 6 h, spleen remained enhanced; both normalized by 24 h, indicating Fe^3+^-MNP clearance. No acute toxicity or morphological changes up to 200 μg/mL within 24 h.	[39]
Natural Melanin Nanoparticles (MNP-Mn)	MNP-Mn^2+^: 3.2	In vitro & In vivo: 3T clinical MRI	MNP-Mn: 18.86 mM^−1^ s^−1^ (3 times as high as that of commercial gadodiamide (6.00 mM^−1^ s^−1^, a clinically used MRI contrast agent)	Cytotoxicity (in vitro): MNP-Mn showed minimal toxicity in Hep-2 and NIH 3T3 cells at up to 800 μg/mL Mn (24 h, MTT assay), reflecting melanin’s good biocompatibility.	Peak liver/kidney contrast at 24 h (cleared by Day 5) and tumor peak at 3 h (cleared by 12 h). No tissue damage or toxicity observed over 30 days.	[43]
Natural Melanin Nanoparticles (RGD-functionalized (biomolecule) PEG-MNPs)	MNP: 4.5 ± 0.5 MNP-PEG: 7 MNP-PEG-Fe: 8.9 MNP-PEG-Fe-RGD: 10.7	In vitro & in vivo: 1T MRI	MNP-PEG-Fe-RGD: 1.2 mM^−1^ s^−1^	Cytotoxicity (in vitro): PEG-MNPs (≤3.125 μM) showed ~90–110% cell viability in NIH-3T3 and U87MG cells after 24 h (MTT assay), indicating high biocompatibility and low cytotoxicity.	U87MG tumor MR signal increased 30% at 4 h (1.25–20 μMFe). Cu-RGD-PEG-MNP64 tumor uptake rose over time: 4.75 ± 0.63% (2 h) →5.87 ± 0.87% (4 h) →5.93 ± 0.89% ID/g (24 h). At 24 h, organ uptake was moderate in liver (15.78 ± 2.55% ID/g) and lower in kidneys (5.34 ± 0.62% ID/g), with clearance mainly via the hepatobiliary route.	[42]
Melanin-Like Nanoparticles (PheoA-MNPs: Pheophorbide A-poly(L-Dopa))	MNP: 140 ± 10 nm MNP-PheoA: 140 ± 10 MNP-PheoA-DOX: 120	9.4T animal MRI system	MNP-Mn^2+^: 17.88 mM^−1^ s^−1^	Cytotoxicity (in vitro): MNP and MNP-PheoA (0–100 µg/mL, 24 h) showed no significant toxicity in HCT116 and HEY-T30 cells, confirming good cytocompatibility as nanocarriers.	PheoA-MNPs + 670 nm NIR induced apoptosis via PDT. DOX/PheoA-MNPs + 808 nm NIR caused greater tumor damage due to combined chemo-photodynamic effects.	[48]

**Table 3:** PTT/PDT/Drug Delivery Applications with Melanin Nanoparticles-Based Systems.

PTT/PDT/Drug Delivery	In Vitro Cell	Concentrations of Photosensitizers	NIR Laser Irradiation in Wavelength (nm)/Power (W/cm^2^) & Duration (min)	Temp. Increase (°C)	Tumor Cell Viability/ Photothermal Efficiency	Drug/Max Loading	Drug Release/ Excretion	Ref.
PTT	Human laryngeal cancer Hep-2 cell lines & NIH 3T3 fibroblasts	In vitro: 1 mL of MNP-Mn aqueous solution at 50, 100, 200 and 400 μg mL^−1^ concentrations	In vitro: 808 nm NIR at 1 to 3 W/cm^2^ for 5 min. In vivo: 808 nm NIR at 2 W/cm^2^ for 5 min.	32.1 °C for 50 μg mL^−1^ 37.1 °C for 100 μg mL^−1^ 46.4 °C for 200 μg mL^−1^ 57.5 °C for 400 μg mL^−1^	In vitro: <40% viability of Hep-2 cells at 200 μg mL^−1^, confirming strong photothermal activity of MNP-Mn. In vivo: MNP-Mn (1.5 mg mL^−1^, 50 μL) with NIR laser treatment raised tumor temperature to 57.5 °C after 3 h, effectively ablating tumors.	*	*	[43]
PTT + PDT+ Drug Delivery	Human colorectal carcinoma cell lines. (HCT 116 cells)	In vitro/in vivo: 0–200 µg mL^−1^	For PTT: In vitro/in vivo: 808 nm NIR at 1.2 W/cm^2^ for 20 min/30 min. For PDT: In vitro/in vivo: 670 nm NIR at 150 mW/cm^2^ for 10 min.	For PTT: 52 °C for 200 μg mL^−1^	Photothermal efficiency: 87.65% for 200 µg mL^−1^.	Max loading of DOX: 35%	Max release of DOX: 23% after 30 min of NIR irradiation.	[48]
PTT	4T1 and HeLa cells	In vitro: 200 μg mL^−1^	In vitro: 808 nm NIR at 2 W/cm^2^ for 5 min.	33.6 °C for 200 μg mL^−1^	In vitro: < 40% of 4T1 cell viability for 200 μg mL^−1^ Photothermal conversion efficiency: 40%	*	*	[44]
PTT	PC-3 cancer cells.	In vitro: 10 μg mL^−1^	In vitro: 808 nm NIR at 464 mW/cm^2^ W/cm^2^ for 5 min.	57 °C for 1.2 mg mL^−1^ of Gd-Mel@SiO_2_	In vitro: 58% suppressed PC-3 cell viability for 10 μg mL^−1^ Heat transduction efficiency: 53%.	*	*	[46]
PTT + Drug Delivery	HeLa cells	In vitro: 200 μg mL^−1^	In vitro: 808 nm NIR at 1.31 W/cm^2^ for 6 min.	30 °C for 100 μg mL^−1^	PDAs@CP3: 28.4% PDAs@CP3-DOX: 17.2%	Max loading efficiency of DOX: 2.562 mmol g^−1^	Max release of DOX: 50% after 55 h in pH 5.0, at 50 °C.	[56]
PTT + Drug Delivery	A549 cells & LLC cells (lung cancer cells)	In vitro: 268.2 nM for A549 395.7 nM for LLC cells	In vitro: 808 nm NIR at 2 W/cm^2^ W/cm^2^ for 10 min.	49.7 °C for 1 mg mL^−1^ of CPT NPs	The apoptosis rate of A549 cells is 26.5% for 268.2 nM. The apoptosis rate of LLC cells is 26.7% for 395.7 nM.	Max loading efficiency of CPT: 66.6%	42.3% of CPT was released in pH 7.4 buffer. 78.5% of CPT in pH 5.0 with NIR irradiation (2 W∙cm^−2^, 5 min).	[45]
PTT	KB cells & 293 cells	In vitro: 75 μg mL^−1^ of 51 nm size CuPDA	In vitro: 808 nm NIR at 3.5 W/cm^2^ for 20 min.	42.2 °C for 75 μg mL^−1^ 51 nm CuPDA	In vitro: Cell viability < 20% at 3 W/cm^2^ laser power density; photothermal efficiencies for 51, 119, and 188 nm NPs were 54.8%, 45.7%, and 25.5%, respectively. In vivo: Tumors fully eliminated after 4 days of 808 nm laser (0.33 W/cm^2^, 20 min) with no recurrence up to 16 days; mice maintained normal body weight post-treatment.	The loading efficiency was 10%, with Cu^2+^ ions released at pH 7.4.	CuPDA NPs have an outstanding tumor retention rate, reaching as high as 8.2% ID/g.	[41]
PTT	HepG2 (human liver cancer) cells	In vitro: 2.0 mg mL^−1^	In vitro: 808 nm NIR at 1 W/cm^2^ for 5 min.	40.3 °C for 2 mg mL^−1^	In vitro: The cell viability decreased to 42% at 100 µg mL^−1^ photothermal conversion efficiency: 13.32%	*	*	[51]

* Value is blank/not available.

## Data Availability

No new data were created or analyzed in this study. Data sharing is not applicable to this article.
